# Explaining consumer suspicion: insights of a vignette study on online product reviews

**DOI:** 10.1007/s12525-022-00549-9

**Published:** 2022-06-06

**Authors:** Tim Kollmer, Andreas Eckhardt, Victoria Reibenspiess

**Affiliations:** 1 Universität Innsbruck, Universitätsstraße 15 6020, Innsbruck, Austria; 2 Washington State University, Carson College of Business, Pullman, WA USA

**Keywords:** Product reviews, Online purchasing behavior, Deception, Suspicion, Vignette study, M1

## Abstract

As part of the online product and service selection and purchase process, many consumers consult and rely on online product reviews. In order to persuade potential customers to buy their products, many organizations and businesses post deceptive product reviews of their own products on their own or third-party websites to their advantage. This creates consumer suspicion about the authenticity and veracity of online product reviews. To better understand how consumers’ experiences of having been deceived by deceptive online product reviews in the past and the density of deception characteristics in an online product review influence their level of suspicion about the review and, ultimately, their intention to buy the product, we conduct a 3 × 3 vignette study. Our results indicate that deceptive characteristics in online product reviews and prior encounters with deception in online marketplaces increase consumer suspicion. Furthermore, we show that preference for a specific product decreases consumer suspicion about reviews of that product. Lastly, we demonstrate that consumer suspicion towards a product decreases purchase intention.

## Introduction

The volume and variety of products and services available for purchase online has increased dramatically in the last ten years, further exploding during the COVID-19 crisis when national governments mandated that non-essential traditional brick-and-mortal retailers remain closed during periods of lockdown. Unlike in brick-and-mortar retail shops, consumers cannot see and touch tangible products on the Internet. Many consumers rely on such reviews as a source of information about the characteristics, quality, and reliability of products. The reviews shape their opinion about products and influence their purchasing decisions (Luca & Zervas, 2016). As a detrimental consequence, the number of vendors trying to manipulate this information channel with deceptive product reviews increases (Luca & Zervas, 2016). Deceptive product reviews are written to lead the reader to a false belief or conclusion about the product (Buller & Burgoon, 1996; Yoo & Gretzel, 2009). The prevalence of deceptive product reviews is very high. In 2004, Amazon unintentionally revealed the identities of anonymous product reviewers, exposing numerous book authors who had rated and recommended their own books on the platform (Luca & Zervas, 2016). Moreover, recent media coverage and practitioner studies report that “unknown” brands of digital devices receive thousands of unverified reviews on Amazon without evidence that the reviewer had purchased or even used the product (Woollacott, 2019). Despite efforts by online merchants to ensure the authenticity of product reviews and combat fraud, an estimate of 25% to 30% of all product reviews are deceptive (Martinez-Torres & Toral, 2019). For instance, recent studies indicate that up to 30% of the reviews on Amazon retail websites and more than 50% of the reviews at Walmart.com are deceptive (Luca & Zervas, 2016; Picchi, 2019).

Whereas scholars rely primarily on algorithms and expert opinions to identify deceptive product reviews (Jindal & Liu, 2008; Plotkina et al., 2020), less is known about what leads consumers to suspect that a product review is deceptive (Zhuang et al., 2018). In this context, we define consumer suspicion as their uncertainty about whether product reviews are true (Buller & Burgoon, 1996; Plotkina et al., 2020), which is driven by their perception of what we refer to as deceptive characteristics (Bart et al., 2005; Zhuang et al., 2018). Extant research into consumer perceptions fails to consider qualitative aspects of the product reviews, such as their content, lexical characteristics, and syntax (Ong et al., 2014; Reyes-Menendez et al., 2019; Zhuang et al., 2018), even though scholars have shown that the lexical, syntactic, and semantic structure of text impacts consumers’ perceptions (Liu et al., 2019). This study contributes to filling the significant knowledge gap about the implications of consumers’ perceptions of whether or not product reviews are deceptive (Ansari & Gupta, 2021; Ghose et al., 2014; Kwark et al., 2014; Song et al., 2019). Our core research objective is to better understand consumer suspicion of product reviews and how this suspicion impacts their intention to purchase the product. Accordingly, we pose the following research question:

- **RQ:** What are the drivers and consequences of consumer suspicion towards online product reviews?

To answer this question, we conduct a vignette-based online experiment using a between-subjects design to identify the factors that influence consumers’ online purchase intention (Atzmüller & Steiner, 2010). We contribute to the information systems (IS) literature on deceptive product reviews and their associated effects in three different ways. First, we show that an increasing density of deceptive characteristics in online product reviews increases consumer suspicion. In doing so, we enhance our understanding of how consumers evaluate online product reviews based on content, language, and syntax (Luan et al., 2016; Zhuang et al., 2018). Second, our results demonstrate that preference for a specific product reduces consumer suspicion of reviews of that product. This expands the dominant concept of consumer suspicion beyond the role of retailer branding and reputation (Kim & Choi, 2012). Third, we show that consumer suspicion significantly negatively affects purchase intention.

The remainder of this paper is structured as follows. First, we synthesize extant research relevant to consumer deception detection and the deceptive characteristics of product reviews. Then we introduce our research model and the corresponding hypotheses. After outlining our research methodology, we analyze the findings of our vignette-based online experiment and evaluate our hypotheses. Finally, we discuss our findings and outline their implications for future research and practice.

## Research background

### Types of deceptive product reviews

The topic of deceptive product reviews has attracted increasing attention in research (Zhuang et al., 2018) and practice (Lappas et al., 2016) in recent years. Scholars refer to such product reviews by various names, including deceptive, misleading, fraudulent, fake, and manipulative (Dellarocas et al., 2007; Kyungmin et al., 2018). In this study, we use the term deceptive. Buller and Burgoon (Buller & Burgoon, 1996) define deception as a message knowingly distributed by a sender to create a false conviction or impression. Yoo and Gretzel (2009) transfer this definition to the field of online communication (Buller & Burgoon, 1996; Yoo & Gretzel, 2009). We rely on both Buller and Burgoon (1996) and Yoo and Gretzel (2009) and define deceptive product reviews as “*product reviews written to lead readers to a false belief or conclusion about a product.”* Scholars have identified various ways that organizations and vendors manipulate product ratings through deceptive product reviews (Wu et al., 2019). There are two basic types of deceptive product reviews. Good-mouthing is the positive manipulation of deceptive reviews of one’s own products to increase consumers’ intention to buy the product. Bad-mouthing, in contrast, is the negative manipulation of deceptive reviews of competitors’ products. Since good-mouthing directly impacts the intention to purchase the targeted products, it dominates deceptive product reviews (Chevalier & Mayzlin, 2006). In this study, we focus on good-mouthing deceptive product review practices, primarily including posting one’s own deceptive reviews or incentivizing others, often monetarily, to write favorable deceptive reviews (Dellarocas & Narayan, 2006). To avoid consumer suspicion, deceivers strive to avoid detection (Zhang et al., 2016) by making their deceptive product reviews appear genuine and by continuously improving the quality of their deceptive product reviews based on past experiences (Krishnan & Wan, 2021). One way to make a deceptive product review appear genuine is to adopt linguistic characteristics similar to authentic reviews, but certain differences between authentic and deceptive product reviews remain detectable (Banerjee & Chua, 2017).

### Detecting a deceptive product review

Studies show that people have limited abilities to detect deception in written statements and transcripts of accounts (Landry & Brigham, 1992; Toma & Hancock, 2012). For example, Kraut and Poe’s (1980) investigation of how accurately human observers can detect a lie reports a detection accuracy of only approximately 57%, i.e., just moderately above the level of chance. Our understanding of deceptive product review detection builds on interpersonal deception theory (IDT) (Buller & Burgoon, 1996). By considering the perspectives of sender and receiver in the information exchange process, IDT shows how individuals deal with deception in face-to-face communication. While the sender wants the receiver to accept the deceptive information as truth, the receiver tries to detect deception based on his own assessment of the credibility of the information. The sender’s success depends on how well the receiver assesses the credibility of the information and detects the deception. Buller and Burgoon (1996) find that receivers tend to have a so-called “truth bias”, which paves the way for misperception and causes them to overlook deception characteristics (Buller & Burgoon, 1996; Plotkina et al., 2020). In face-to-face interactions, consumers detect deception via facial expression, tone of voice, and body gestures. (D. Zhang et al., 2016). In contrast, consumers rely on different cues to detect deception in online product reviews, such as the rhetorical style of the product review (Banerjee & Chua, 2014; Zhang et al., 2016).

When consumers detect deception, they become suspicious, which Buller & Burgoon (Buller & Burgoon, 1996, p. 205) define as “uncertainty whether the sender is telling the truth or lying.” Consumer suspicion can be induced by making them aware of deceptive tactics and training them to guard against deceptive tactics in product reviews Oza et al., 2010). Some studies show that experience is an important factor influencing deception detection (Hartwig et al., 2004; O'Sullivan & Ekman, 2008). Consumers who have previously experienced online fraud are more likely to question the veracity and accuracy of information provided by product reviews (Zhuang et al., 2018). To protect their consumers, some online marketplaces have established mechanisms to prevent deceptive product reviews. In 2009, Amazon introduced the verified purchase badge to ensure that the reviewer purchases the product before reviewing it (He et al., 2020). In addition, some online marketplaces allow consumers to rate how helpful they find a review, which should spotlight authentic reviews (Mudambi & Schuff, 2010). Despite such mechanisms, the problem of deceptive product reviews in online marketplaces persists (Luca & Zervas, 2016). Most IS research into deceptive product reviews focuses on perceived review credibility as the counterpart of suspicion. Consumers assess the meta-data of reviews to evaluate whether or not they are credible, and they assess reviewers’ reputations based on available profile cues (Bae & Lee, 2011; Cheung et al., 2012; Lim & van der Heide, 2015). Consumers also evaluate the content of reviews, including the quality and degree of one-sidedness of the information and argument to assess its credibility (Cheung et al., 2012; Shan, 2016). Adding positive product reviews increases consumer suspicion while removing negative product reviews decreases consumer suspicion (Zhuang et al., 2018). Consumers’ truth bias is amplified by their product preferences since it reduces their cognitive attention through arousal and pleasure (LaRose, 2001; Luan et al., 2016). In conclusion, extant studies examine factors that influence credibility but fail to investigate the connection between the lexical, syntactic, and semantic structure of reviews and consumer suspicion.

### Characteristics of deceptive product reviews

Deceptive product reviews differ from authentic product reviews in several ways (Vallurupalli & Bose, 2020; Yin et al., 2021). Scholars have developed several complex algorithms and machine learning models to detect deceptive product reviews automatically (Fuller et al., 2011, 2013). These algorithms and machine learning models incorporate quantitative and qualitative review patterns such as the review history of the reviewer, similarity across different product reviews by the reviewer, and temporal patterns of the product reviews (Jindal & Liu, 2008; Zhou et al., 2013). Such algorithms and machine learning models significantly surpass consumers’ ability to detect deceptive product reviews, achieving an accuracy rate of up to 89% (Krishnan & Wan, 2021). In contrast, consumers primarily rely on the review content to identify deception (Barbado et al., 2019; Hu et al., 2014; Zinko et al., 2020). Previous studies have identified patterns within deceptive product reviews that can be classified into four concepts*:* Comprehensibility, specificity, exaggeration, and negligence (Banerjee & Chua, 2017; Plotkina et al., 2020). Each concept is comprised of two to three deceptive characteristics that provide cues whether a review is deceptive or authentic (Hu et al., 2011; Zhuang et al., 2018).

Comprehensibility is the extent to which a product review is understandable. Banerjee and Chua (2017) find that the titles of authentic reviews are longer than those of deceptive reviews. In addition, deceptive and authentic product reviews show deviations in their text length. The length of the text, for example, varies within a certain interval, so that central information and required emotions can be presented in the shortest possible manner (Ott et al., 2011). Yoo and Gretzel (2009) show that deceptive product reviews with an average word count of 164 are 38 words shorter on average than authentic product reviews. Furthermore, the simplicity of the product description in a review is a further identification criterion for deception. To make the information contained in product reviews accessible to the broadest possible range of consumers, they often employ a simpler syntax than authentic reviews (Büschken & Allenby, 2016; Hu et al., 2012).Specificity is another important conceptual pattern common to deceptive product reviews*.*

 Specificity refers to all factors related to the details of the product being reviewed (Banerjee & Chua, 2017; Tausczik & Pennebaker, 2010). One specificity-related characteristic is whether and how frequently the brand name is mentioned. While only 62.5% of authentic product reviews mention the brand name of the reviewed product, almost every deceptive product review includes the brand name (90.5%). Furthermore, deceptive product reviews are twice as likely to mention the brand name more than once than authentic product reviews (Yoo & Gretzel, 2009). In addition, deceptive product reviews contain fewer temporal words (e.g., “today,” “yesterday.” and “tomorrow”) (Banerjee & Chua, 2017).

A third conceptual pattern common to deceptive product reviews is exaggeration, which is employed to initiate psychological processes and evoke personal emotions in consumers (Banerjee & Chua, 2017). Deceivers exaggerate by focusing on sentiment content in the text to evoke strong positive feelings and increase the intention to purchase (Ott et al., 2011). The titles and texts of authentic reviews include fewer exclamation marks than those of deceptive reviews (Banerjee & Chua, 2017; Zhang & Peng, 2015).

The conceptual pattern of negligence includes textual indicators that coincidentally leak out in a review. Deceptive product reviews contain more self-references, i.e., the total number of pronouns in the first person singular and plural (I, me, mine / we, us, ours), than authentic product reviews (Ott et al., 2011; Tausczik & Pennebaker, 2010). On average, deceptive product reviews contain 6.49 pronouns. This means that the number of self-references, despite shorter text length, is 27% higher in deceptive than in authentic product reviews (Yoo & Gretzel, 2009). In order to measure the effect of the conceptual patterns of comprehensibility, specificity, exaggeration, and negligence, we count the iterations of the deceptive characteristics that comprise them in online product reviews.

The more deceptive characteristics that occur in a product review, the higher the density of deceptive characteristics the review has (see Appendix C for an overview of the deceptive characteristics).

## Research model and hypotheses development

Building on the work of Buller and Burgoon (1996) and Ott et al. (2011), we explain how consumers become suspicious and discuss the enablers (e.g., the density of deceptive characteristics and deception experience) and inhibitors (product preference) of consumer suspicion in the online product review context.

Consumer suspicion increases when there is a deviation from expectations or norms (Ahluwalia & Burnkrant, 2004). To verify this suspicion, consumers rely on cues to assess the salience of this deviation (Ahluwalia & Burnkrant, 2004) and on cues to detect deception (Friestad & Wright, 1994; Plotkina et al., 2020). Biswas et al. (2021) show that when consumers recognize cues in product reviews, like negative emotions, their perception of the review is impacted. Prior research also indicates that characteristics of the online marketplace and its products, for instance, brand strength, affect consumer suspicion (Bart et al., 2005). The density of these characteristics differs between authentic and deceptive product reviews and provides consumers cues for deception detection. Previous studies show that an increasing number of manipulative cues leads to an increased probability of deception (Hu et al., 2011; Zhuang et al., 2018), and the more deceptive characteristics that occur in product reviews, the greater their density. As a result, we derive the following hypothesis:

- **H1:** The higher the density of deceptive characteristics in product reviews, the greater the consumer suspicion of product reviews.

In face-to-face communication, deceivers rely on specific tactics such as verbal expressions to convince others. Individuals can be sensitized to those cues in order to detect deception more accurately (Ekman & Friesen, 1969; Liu et al., 2019). Correspondingly, individuals exposed to those cues in the past may more easily recognize them as being deceptive. For example, Mann et al. (2004) find that experienced police officers conducting interrogations had better results in detecting suspects’ lies in a highly familiar context. Similarly, research shows that experienced evaluators are better at detecting deception than less experienced evaluators (Hartwig et al., 2004; O’Sullivan and Ekman, 2008). In addition, experienced evaluators are more skeptical than non-experts and are less inclined to believe that other people acting online are truthful. Thus, consumers who are experienced with deception in online reviews regard product reviews more skeptically than consumers who lack experience with such deception (Reinhard et al., 2013). Hence, we hypothesize:

- **H2**: The higher the prior deception experience, the higher the consumer suspicion of product reviews.

While environmental characteristics in online marketplaces can help experienced consumers detect deceptive information in product reviews, they also create stimuli that invoke emotional reactions (affective mechanism) and different beliefs, thoughts, or perceptions about products and brands (cognitive mechanism) (Xiao & Benbasat, 2011). An example of such a stimulus is consumer product preferences. Consumers who prefer a certain product assign greater value to that product and are more likely to exhibit a positive attitude toward it. Consumers who prefer a certain product also experience more arousal and pleasure towards the product, which can diminish their self-control (LaRose, 2001) and reduce their cognitive attention toward deceptive product-related information (Luan et al., 2016). Emotionally involved consumers are thus less likely to rely on other information characteristics, such as the credibility of the information source when judging a product review for a product they prefer (Zhang & Watts, 2016). Thus, we hypothesize:

- **H3**: The higher the product preference, the lower the consumer suspicion of product reviews.

Consumers have more confidence in the judgment of a source they consider highly credible (Luo et al., 2013). While information characteristics are attained through prior deception experience (i.e., product-related anomalies), they can also lead to the conjecture that the content and credibility of a product review is suspicious (Xiao & Benbasat, 2011). Thus, if consumers perceive a product review as suspicious, they may be reluctant to adopt the viewpoint of the reviewer and thus develop less positive attitudes towards the product. A product review from a less credible source (e.g., unknown platform user) can even strengthen consumers’ negative judgment of the product (Luo et al., 2013) and even dissuade them from buying a product they would otherwise purchase (Anderson & Simester, 2014). Accordingly, we hypothesize:

- **H4**: The higher the consumer suspicion of product reviews, the lower their purchasing intentions.

**Fig. 1:**
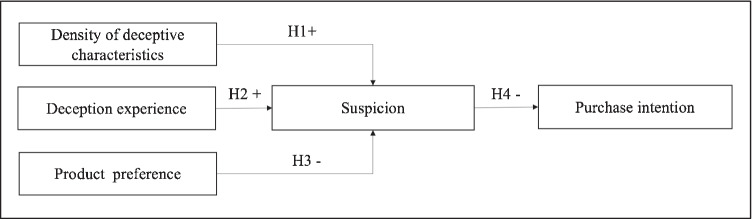
Research model

Figure 1 depicts our resulting research model, which hypothesizes that deception experience and the density of deceptive characteristics positively influence consumer suspicion (Hypotheses 1–2), while product preference reduces it. In turn, increased suspicion reduces consumers’ intention to purchase (Hypothesis 4).

## Methodology

### Vignette study

Since consumer suspicion of deceptive product reviews is difficult to observe, a vignette study is an appropriate tool for testing our hypotheses. Since vignette studies provide absolute control over the independent variables, vignettes can be used to create hypothetical scenarios (Gould, 1996). In our online vignette-based experiment (Aguinis & Bradley, 2014), we examine and isolate the causal mechanisms of consumer perceptions and how they affect their suspicion of product reviews (see Appendix A for more details).

Our vignette-based experiment study design also includes a parallel survey to measure additional characteristics following the vignette study. Hypothetical scenarios work best if participants behave as realistically as possible (Lohrke et al., 2010). To elicit realistic behavior, we chose the highly popular FIFA World Cup as the context of this study. Using a filter question, we divided participants into groups according to their soccer affinity, which we equated with participants’ potential interest in purchasing soccer merchandising products. Our vignette-based study adopts a variation of the between-person approach, such that all participants are exposed to several vignettes (Atzmüller & Steiner, 2010).

### Study design

To minimize participants’ predisposition to suspicion, we recruited participants from the German-speaking region in Europe (Germany, Austria, Switzerland) by posting an invitation to participate in an “online study about product reviews” on social networks. We then contacted respondents by email. Of the 190 participants who started the online experiment, we excluded 35 who did not have an affinity for soccer or who failed the manipulation checks. Our final sample thus included N = 155 participants (97 males and 58 females). 66% of the participants were between 21 and 30 years old, 24% percent were between 31 and 40 years old, and 10% were over 40 years old. Over 90% of the participants had previous experience with online shopping and over 30% wrote their own product reviews at least once a month. About half of the participants reported prior experience with deception in online marketplaces, and nearly all of the participants described the vignette setting as realistic in the post-experiment questionnaire.

**Fig. 2:**
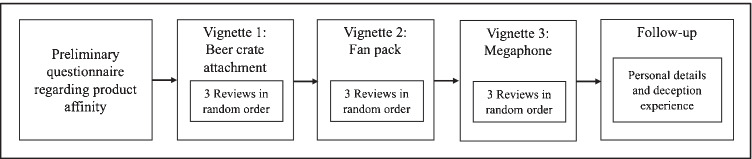
Study design

To ensure widespread product affinity among the participants, all three vignettes related to purchasing a FIFA World Cup-related product. To reduce bias, we did not reveal our focus on deceptive product reviews or suspicion. In the vignettes, three best-selling FIFA World Cup merchandise products were for sale: a branded beer crate table-top attachment, a pack of branded fan merchandise, and a branded megaphone. All three products were in the medium price range, between 19 and 37 euros, and had a similar number of reviews and average ratings (see Appendix A). Participants were shown three carefully created scenarios (vignettes) describing one of the World Cup merchandizing products, the task, and the situation (Hu et al., 2012; Ott et al., 2011; Sun, 2012). The first step of each vignette included a query of the participants’ product affinity. In the second step, the participants were shown three product reviews with three different densities of deceptive characteristics (low, medium, and high) in random order. E-commerce experts selected the reviews for each vignette to ensure that different patterns of deceptive characteristics occur in a realistic intensity. In addition, the heterogeneity in the density of deceptive characteristics was validated through a check using ReviewMeta, which is a specialized tool for identifying deceptive product reviews that considers many characteristics of deceptive product reviews that overlap with our study (Noonan, 2021). ReviewMeta has been used both in research and in practice to detect deceptive product reviews (Petrescu et al., 2018). After reading the vignette, participants could access the product reviews. In each vignette, participants were shown three reviews, one at a time and in random order. After reading each review, participants reported their purchase intention and level of suspicion. After having completed these steps for all three reviews, they began with the next vignette (see Fig. 2).

In a pilot study, ten participants completed a preliminary questionnaire to verify the contextual applicability of all items and the comprehensibility of the vignette scenario. In the main vignette study, we asked participants to rate the degree to which each product review raised suspicion or impacted their purchase intention on a five-point Likert scale (Cheung et al., 2012). In each of the three vignettes, we tested three product reviews for their deceptive characteristics (see Appendix C). Hence, each participant read three vignettes and nine reviews. All validated measurement items, vignettes, and reviews are found in Appendices A, B, and D.

In preparation for the study, we counted the number of deceptive characteristics in each online product review that exceeded a critical value to measure the density of deceptive characteristics. We set the critical values based on extant quantitative metrics of deception and authenticity in product reviews (Banerjee & Chua, 2017; Büschken & Allenby, 2016; Li & Zhan, 2011; Yoo & Gretzel, 2009). For example, Yoo and Gretzel (2009) found out that deceptive product reviews are, on average, 19% shorter than authentic reviews. Hence, we identify shorter text length as a cue or characteristic of deceptive product review and, following the authors’ lead, set the critical value for average text length at 164 words. We then divided the reviews into three groups based on the number of deceptive characteristics above the critical value, i.e., the level of deception (e.g., Banerjee & Chua, 2017; Büschken & Allenby, 2016; Yoo & Gretzel, 2009). We classified reviews with three or fewer deceptive characteristics as low density, reviews with four or five deceptive characteristics as medium density, and reviews with six or more deceptive characteristics as high density.

During the study, participants required, on average, about 14 min to participate in our study, between 42 and 56 s to consider each vignette, and about 51 s, consistently, to evaluate each review. In a follow-up survey, we asked participants for demographic information, about their previous experience with deceptive online product reviews, and how realistic they considered the vignettes. We included these variables and demographics as covariates to isolate the effects of the manipulated variables. We also performed manipulation checks to determine whether the participants understood the vignette experiment, dropping participants from the sample who failed the checks.

## Data analysis and results

### Reliability tests

We conducted two reliability tests using SPSS Statistics 25. Applying the split-half method, we tested the reliability of our study by randomly dividing our data into two random subgroups. The calculation of the reliability coefficient yields values between 87% and 90% of consistent measurements for both groups for each iteration (Chau, 1999). We therefore concluded that the major constructs of our study are sufficiently reliable. In addition, we calculated Cronbach’s alpha to ensure the internal consistency of our scales (Cortina, 1993). Cronbach’s alpha ranges from 0.6 to 0.95 (Lyberg et al., 1997) indicate internal consistency and the lack of undesired redundancy between the different items of the scale (Diamantopoulos et al., 2012; Drolet & Morrison, 2001). The tested constructs had acceptable Cronbach’s alpha values between 0.679 and 0.915. Table 1 shows the Cronbach’s alpha values for the purchase intention and suspicion among the reviews (R1-R9).

**Table 1:** Cronbach’s Alpha values for purchase intention and suspicion

	Beer crate attachment			Fan pack			Megaphone		
	R1	R2	R3	R4	R5	R6	R7	R8	R9
Cronbach’s Alpha: Purchase Intention	0.855	0.875	0.845	0.909	0.890	0.915	0.846	0.709	0.679
Cronbach’s Alpha: Suspicion	0.730	0.757	0.725	0.880	0.801	0.834	0.787	0.677	0.738

The results of both tests confirm the reliability of the constructs purchase intention and suspicion. Therefore, we continued with further analysis.

### Hypotheses testing

Overall, our objective was to test whether the characteristics of the deceptive product reviews lead to measurable differences between the deceptive and authentic product reviews. Due to the non-parametric nature of our data and because we have a connected sample, we use the Friedman test to examine H1 (Demšar, 2006) and the Wilcoxon test to examine H2 (Altman, 1997). We ran a regression analysis to evaluate H3 and H4 (Green & Salkind, 2012). The Friedman test determined the degree to which participant suspicion is driven by a low, medium, or high density of deceptive characteristics. We find a significant difference regarding the density of deceptive characteristics, X2 (2) = 21.109; *p* < 0.001. Our results show a significant difference for reviews with a medium and high density of deceptive characteristics on consumer suspicion compared to those with a low density. However, we find no significant difference between participants’ suspicion about reviews with a medium vs. reviews with a high density of deceptive characteristics. We applied the Dunn-Bonferroni test to reveal differences between the participants’ suspicion regarding product reviews with low density and product reviews with medium density (*p* < 0.001) and product reviews with high density (*p* < 0.002). The results are displayed in Table 2 in the column labeled *adjusted significance*. There were no significant differences between any other variables. In addition, Kendall’s W is 0.68, indicating a large effect size and a good agreement between subjects. Thus, our results confirm H1.

**Table 2:** Dunn-Bonferroni test: pairwise comparisons

Suspicion level relative to density	Test statistic	Std. error	Std. test statistic	Sig.	Adj. sig.
Low density – High density	0.410	0.114	3.607	0.000	0.001
Low density – Medium density	0.432	0.114	3.805	0.000	0.000
High density – Medium density	−0.023	0.114	−0.199	0.842	1.000

Table 3 shows the signed-rank sums of the suspicion levels of participants with and without deception experience. Suspicion of the product reviews has 39 negative ranks, 28 positive ranks, and four ties. While participants with deception experience are somewhat less suspicious than participants without deception experience, the test statistics show no significant difference between the two variables, as the asymptotic significance of both is above the target significance level of 0.05. Overall, the descriptive statistics and the results of the Wilcoxon test show that there is no demonstrable difference between prior deception experience and participants’ suspicion. Therefore, our results do not support H2.

**Table 3:** Wilcoxon Test: signed-rank distribution of suspicion

Suspicion with deception experience – Suspicion without deception experience	N	Mean rank	Sum of ranks
Negative ranks	39	34.35	1339.50
Positive ranks	28	33.52	938.50
Ties	4		
Total	71		
Z			−1.253

We examined H3 to evaluate to what extent the preference for one of the products in the vignettes (beer crate attachment, fan pack, megaphone) influences participants’ suspicion of product reviews. We conducted regression analyses between the product preferences and the corresponding suspicion. Table 4 summarizes the coefficients and the standard errors. The results show that product preference has a significant negative effect on participants’ suspicion of the reviews for all three products. Therefore, our results support H3.

**Table 4:** Results of regression analyses between product preferences and suspicion

	Product preference: Beer crate attachment	Product preference: Fan pack	Product preference: Megaphone
Suspicion	0.260*** (0.047)		
Beer crate attachment			
Suspicion		−0.283*** (0.052)	
Fan pack			
Suspicion			−0.143*** ^(0.047)^
Megaphone			

Standard errors are in parentheses. ***, **, * indicate significance at the 1%, 5%, and 10% levels

We tested H4 to evaluate whether perceived suspicion of product reviews influences purchase intention. Therefore, we conducted regression analyses between participants’ suspicion of the different product reviews and the corresponding purchase intentions. Table 5 provides the coefficients and standard errors.

**Table 5:** Results of regression analysis between participants’ suspicion and purchasing intention

	Suspicion: Beer crate attachment	Suspicion: Fan pack	Suspicion: Megaphone
Purchase decision	−0.701*** (0.081)		
Beer crate attachment			
Purchase decision		−0.615*** (0.077)	
Fan pack			
Purchase decision			−0.536*** (0.073)
Megaphone			

Standard errors are in parentheses. ***, **, * indicate significance at the 1%, 5%, and 10% levels

The results show that participants’ suspicion has significant negative effects on their purchase intention for all three products. Therefore, the results provide support for H4.

## Discussion

### Conclusion of findings and theoretical implications

There is significant social psychology research into deception detection focusing on identifying when people are lying and applying manipulative practices to influence other people’s behavior (Bond & DePaulo, 2006; Meissner & Kassin, 2002; Plotkina et al., 2020; Reinhard et al., 2013). However, most research in this field focuses on how authorities such as police and law enforcement agencies can tell when suspects are lying. As e-commerce continues along its sharp growth trajectory and as consumers shop online for an even wider range of products, their need to be able to detect deception grows ever more important. In the face of constant information overload, online shoppers need to assess whether product information and product reviews are authentic or deceptive. While some extant research considers users’ perceptions of deceptive product reviews (e.g., Reyes-Menendez et al., 2019; Zhuang et al., 2018), we identify a gap in IS research into how the density of deceptive characteristics in product reviews, as cues of manipulation, affects consumer suspicion and their subsequent purchase intention. To fill this research gap, we conducted an online-based between-subject vignette experiment in which participants were shown three related online product vignettes, each followed by three online product reviews with different densities of deceptive characteristics. In the following, we outline the theoretical and practical implications of our research. As a result of our research approach, we enhance existing research (e.g., Cheung et al., 2009; Kim & Choi, 2012; Zhuang et al., 2018) by the following findings. First, our results show that consumers perceive a medium and high density of deceptive characteristics in online product reviews as cues for deception. Our analysis confirms our hypothesis (H1) that consumer suspicion increases when they detect a threshold level of deceptive characteristics in product reviews. We also expand the scope of research into deceptive product reviews and the factors that influence suspicion and the credibility of product reviews (e.g., Ong et al., 2014; Reyes-Menendez et al., 2019; Zhuang et al., 2018) by providing new insights into consumers’ perceptions of the contextual characteristics of product reviews. In addition, we bridge existing studies examining the contextual characteristics of deceptive and authentic product reviews (Banerjee & Chua, 2017; Yoo & Gretzel, 2009) with research on consumer perceptions of product reviews (Ansari & Gupta, 2021; Zhuang et al., 2018). We demonstrate that an increasing density of deceptive characteristics increases consumer suspicion of product reviews. The relation between deceptive cues and consumer suspicion also intersects with other IS fields, such as IS security. Within security research, the concept of awareness is used to explain an individual’s attention to organizational security efforts (Wolf et al., 2011). The concepts of awareness and suspicion both relate to consumers’ attention to cues of manipulation (Jaeger & Eckhardt, 2021). While a high level of awareness or suspicion protects consumers against deception, a low level of awareness may not have the same effect as a low level of suspicion. While low awareness would lead to cues not being recognized, low suspicion may lead consumers to misjudge cues of manipulation and therefore underestimate the threat of deception. Further research is necessary to investigate the theoretical distinction between awareness and suspicion and the circumstances under which people fail to recognize or misinterpret cues. Second, our study identifies drivers of consumer suspicion of product reviews (i.e., prior online deception experiences, deceptive characteristics of product reviews) and deception detection. Based on extant research identifying experience as an important factor influencing deception detection (Hartwig et al., 2004; O’Sullivan & Ekman, 2008) and showing that consumers with previous online fraud experience are more likely to question the veracity and accuracy of information provided by product reviews (Zhuang et al., 2018), we hypothesized that previous experience with deception in online marketplaces leads to greater suspicion (H2). However, our results do not support this hypothesis and are more closely aligned with studies finding no significant effect of prior experience on judges’ accuracy in deception detection (Bond & DePaulo, 2006). One possible explanation for our unexpected finding is that affective and cognitive mechanisms diminish the effect of consumers’ prior experience and expert knowledge and distort their judgment (Hartwig et al., 2004).

Our study also explores how consumers’ product preferences influence their ability to detect deceptive product reviews. Our results support our hypothesis that product preferences reduce deception detection ability, finding that consumers who prefer a specific product view its product reviews with less suspicion (H3). Our findings thus identify product preference as an important decision determinant and provide richer insights into consumer suspicion of product information.

Third, our results confirm our hypothesis that consumers who are suspicious of a product review find the review less credible, resulting in lower purchase intention (H4). This complements recent research showing that enhanced perceived credibility of a product review increases purchase intention (Grewal & Stephen, 2019). Extant research tends to focus either on the credibility of the product review (Reyes-Menendez et al., 2019) or on consumer suspicion (Zhuang et al., 2018). Since our research points to a bridge between these two similar concepts, we recommend that future research on product reviews examine credibility and suspicion in a single approach with unified determinants and consequences. Specifically, further research is needed to fully understand how suspicion impacts other aspects of and steps in the purchase process, including information gathering and recommending products to other consumers.

### Implications for practice

Based on our findings, we derive actionable recommendations for how online marketplace owners can protect their customers from deceptive product reviews. Although our findings indicate that consumer suspicion increases when the product review has a medium or high density of deceptive characteristics, the average overall suspicion rate is low. Nonetheless, consumer suspicion significantly lowers the intention to purchase the recommended product. To avoid drops in purchase intention and to create customer trust, long-term satisfaction, and loyalty, online marketplace owners need to act and prevent or remove deceptive reviews of their products. We recommend three steps to safeguard the product review process.

First, our results show that consumers do not always recognize the characteristics of deceptive product reviews, so e-commerce owners should create transparency and authenticity features in product reviews. In some online marketplaces, a verified purchase is the sole indicator of product review authenticity (He et al., 2020), and sometimes consumers can rate how helpful they find a product review (Singh et al., 2017). Unfortunately, some organizations exploit these features to promote certain products through deceptive product reviews (Zhu, 2021), driving consumers to use third-party authentication services such as ReviewMeta, which analyzes rating patterns using publicly available data. Our results show that even individuals with online deception experience may not be sensitized to deceptive product reviews. We recommend that online marketplaces rate each review and identify the characteristics that speak for and against its authenticity.

Second, building on research proving the efficacy of proactively training consumers to recognize phishing practices (Jansson & von Solms, 2013), we recommend that online marketplaces train their customers to recognize deceptive product reviews.

Third, since organizations and individuals who generate deceptive product reviews are highly motivated to adapt to environmental changes and improve their methods constantly, we recommend that online marketplaces continuously update and improve their deception recognition algorithms.

### Limitations

Our study is limited in several ways. First, our textual vignettes describe a purchasing process on Amazon. While this approach has been applied in past research, it also has limitations. It is possible that participants, who had negative experiences shopping on Amazon, have frequently encountered deceptive product reviews on Amazon, and therefore expect product reviews there to be deceptive (Aguinis & Bradley, 2014). Second, the study setting was hypothetical and required participants to be active protagonists. Even though we designed the vignettes carefully and pre-tested how realistic, credible, and authentic they are perceived, and even though we controlled for age, prior experience with product reviews, perceived realism of the scenario and self-assessment of ability to participate in the hypothetical setting, participant behavior in hypothetical settings may deviate from their behavior in real-world settings. In a real-world online marketplace product research setting, for example, participants would have the option of interacting more with the online marketplace or with other resources to collect further information about the product of interest. Third, we treated the density of deceptive characteristics as a singular construct without considering possible interference between and among individual deceptive characteristics. Fourth, we identified relevant deceptive characteristics based on previous studies, but other characteristics of the tested reviews may have affected the participants, too. Finally, while setting critical values for each deceptive characteristic to measure density has benefits, it also reduces the impact of outliers for certain values.

## Recommendations for future research

Our results point to several promising avenues of future research. First, our results show that consumers’ product preference inhibits their suspicion about reviews of that product. Future research should test the relevance of this finding in other research domains, such as in the context of phishing attempts. For example, does someone’s preference for a product reduce their level of awareness of the security risks of a phishing attack if the phishing mail is related to the product they prefer? Second, while our research focuses on the perception of qualitative elements of deceptive and authentic product reviews and previous research focuses on the impact of quantitative elements, such as adding and deleting reviews in response to consumer suspicion (Zhuang, 2018), future research should consider how the interplay between and combinations of qualitative and quantitative product review elements, such as star ratings and emotional content, influence consumer suspicion. Third, our results indicate that the overall density of deceptive characteristics influences consumer suspicion of the authenticity of the product review. Future research should also consider how individual characteristics, such as brand referencing, and combinations of individual characteristics of deceptive product reviews, influence consumer suspicion. Finally, as mentioned in the implications section above, building on our finding that consumer suspicion negatively affects purchase intention, further research should investigate how consumer suspicion impacts other aspects of and steps in the entire purchase process, including information gathering recommending the product to other consumers.

## Figures and Tables

**Fig. 3:**
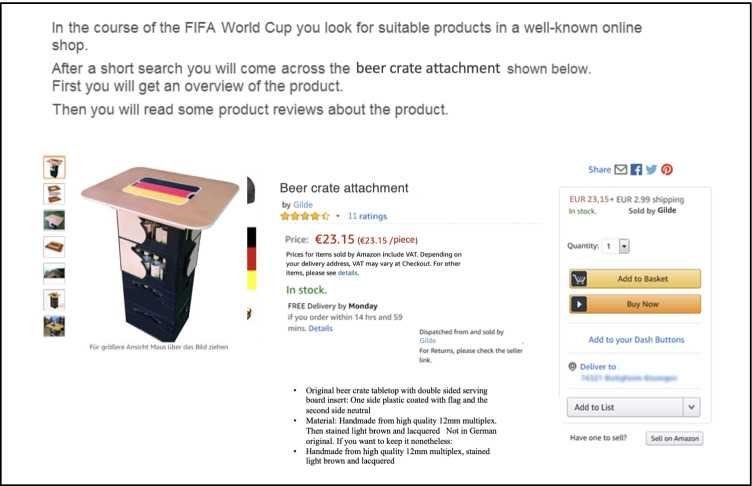
Vignette beer crate attachment

**Fig. 4:**
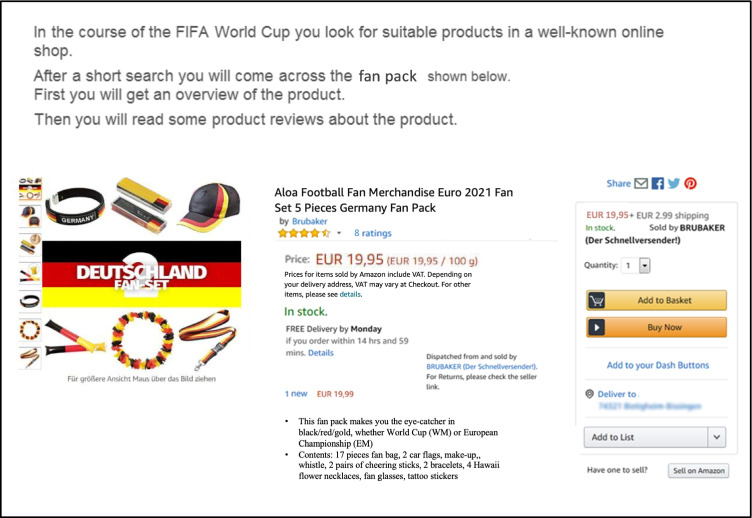
Vignette fan pack

**Fig. 5:**
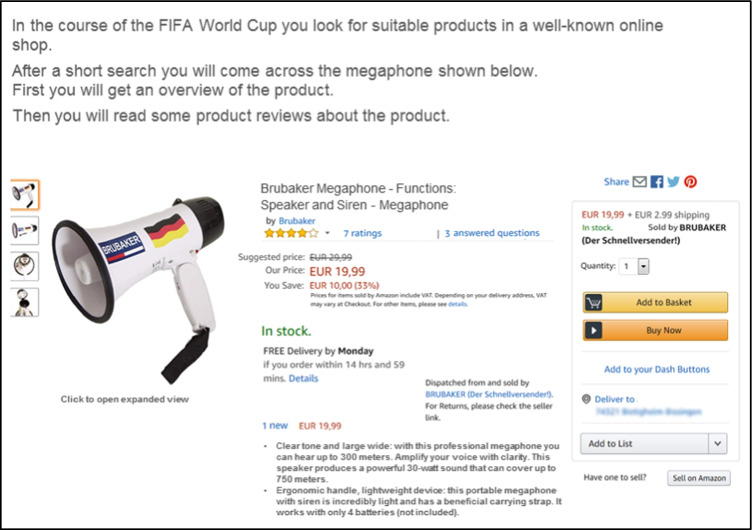
Vignette megaphone

## Appendix A Vignette overview

According to Atzmüller and Steiner (2010), a vignette is a short and carefully constructed description of a person, object, or situation. The information presented by the vignettes enables the participants to assess the chosen situation. To check that the interpretation effect of the vignettes cannot be counteracted by randomization, we conducted a pilot study to validate the degree of realism of the vignettes and the design of the vignette study. The main criterion for the design of vignettes is to make them consistent and plausible so that the participants behave realistically. For this reason, the presentation of the products in the vignettes corresponds to the structure and content of one of the largest online retailers (i.e., Amazon). Also, it is important to structure the vignette with the right level of detail. The vignettes were described in a structured and straightforward manner to ideally introduce the participants to the vignette situation without overburdening them. The vignettes are shown in Figs. 3, 4, 5.

## Appendix B Measurement items

During the vignette study, we collected various responses from the participants. For each vignette, the participants were asked to indicate their respective product preferences (Xiao & Benbasat, 2011). Then we showed each participant three product reviews for each of the three vignettes, one at a time and in random order. The reviews provide different densities of deceptive characteristics. The participants were asked to indicate their suspicion and intent to purchase each product. Lastly, we collected demographics and information about online shopping behavior, reviews experience, and deception experience (see Table 6).

**Table 6:** Measurement items

Item	Wording	Scale	References
Product preference	I like the product.	5-point Likert scale (1 = strongly disagree, 5 = strongly agree)	(Xiao & Benbasat, 2011)
	I would buy the product.		
	I would recommend the product.		
Title length	Critical value: Less than 25 characters		(Banerjee & Chua, 2017)
Text length	Critical value: Less than 164 words		(Yoo & Gretzel, 2009)
Syntax	Critical value: Less than 7 words per Sentence		(Büschken & Allenby, 2016)
Temporal	Critical value: Less than 1 temporal word		(Banerjee & Chua, 2017)
Brand reference	Critical value: Less than 1 brand reference		(Yoo & Gretzel, 2009)
Emotion	Critical value: More than 5% of the content are emotional		(Yoo & Gretzel, 2009)
Exclamation marks	Critical value: At least one exclamation mark is used.		(Li & Zhan, 2011)
Self-referencing	Critical value: More than 4% of the content are self-references.		(Yoo & Gretzel, 2009)
Exclusion words	Critical value: At least one exclusion word is used in the review		(Banerjee & Chua, 2017)
Suspicion	The product reviews are not factual.	5-point Likert scale (1 = strongly disagree, 5 = strongly agree)	(Cheung et al., 2012)
	The product reviews are not credible.		
Purchase intention	The information from the product reviews contributed to my knowledge of the product and helped to make a purchase decision.	5-point Likert scale (1 = strongly disagree, 5 = strongly agree)	(Cheung et al., 2009)
	The product review motivated me to purchase the product.		
	The product review has enhanced my effectiveness in making purchase decisions.		
	The product review made it easier for me to make a purchase decision.		
Age group	Please indicate your age:	≤ 20 years, 21–30 years, 31–40 years, 41–50 years, ≥ 50 years	
Gender	Please indicate your gender:	Female/ male/ diverse	
Internet shopping behavior	How often do you shop on the Internet?	Daily, Several times a week, Weekly, Monthly, Seldom, Never	
Product review expertise	How often do you write product reviews yourself?	Daily, Several times a week, Weekly, Monthly, Seldom, Never	
Deception experience	Have you had any experience with Internet deception in the past?	Yes/no	
Perceived realism	I was able to put myself in the hypothetical setting in the described scenario. The situation in the described scenario was realistic.	5-point Likert scale (1 = strongly agree, 5 = strongly disagree)	

## Appendix C Density of deceptive characteristics

During the study, each participant was shown nine product reviews: three reviews for each of the three vignettes. We conducted the following analysis to determine the density of the deceptive product reviews. Based on a comprehensive literature review, we identified nine characteristics that show significant differences between authentic and deceptive product reviews (Banerjee & Chua, 2017; Büschken & Allenby, 2016; Li & Zhan, 2011; Yoo & Gretzel, 2009). The critical value of deceptive characteristics represents a value where the review is more likely to be deceptive. We defined critical values for all nine characteristics of deceptive product reviews. Furthermore, we analyzed the qualitative measures of all tested reviews. As a result, we counted the number of deceptive characteristics above the critical value to determine the density of deceptive characteristics for the reviews. Table 7 depicts the analysis of the nine product reviews of our study. Moreover, we divided the reviews into three groups. The first group indicates product reviews with three or fewer characteristics above the critical value, indicating a low density of deceptive characteristics. In addition, product reviews with four or five characteristics above the threshold are considered product reviews with a medium density of deceptive characteristics. Lastly, product reviews with more than six characteristics above the critical value are classified as product reviews with a high density of deceptive characteristics. Table 8

**Table 7:** Density of deceptive characteristics

	Item and critical value	Beer crate attachment			Fan pack			Megaphone		
		R1	R2	R3	R4	R5	R6	R7	R8	R9
*Comprehensibility*	Title length (<25 Characters) (Banerjee & Chua, 2017)	27	21	12	26	37	20	26	18	103
	Text length (<164 Words) (Yoo & Gretzel, 2009)	33	62	48	35	29	57	41	37	59
	Syntax (<7 words per sentence) (Büschken & Allenby, 2016)	11	6.20	6.86	5.83	7.25	9.50	6.00	6.50	9.43
*Specificity*	Temporal words (<1) (Banerjee & Chua, 2017)	1	2	0	1	0	0	1	1	0
	Brand reference (≥1) (Yoo & Gretzel, 2009)	0	0	0	0	0	0	0	0	0
*Exaggeration*	Emotion (>5% of words) (Yoo & Gretzel, 2009)	6%	4.8%	2%	8.5%	10.3%	0%	4.8%	5.4%	0%
	Exclamation marks (≥1 per review) (Li & Zhan, 2011)	0	3	2	3	2	3	0	0	1
*Negligence*	Self-referencing (>4% of words) (Yoo & Gretzel, 2009)	3%	4.8%	2.8%	3.4%	1.8%	9.1%	4.8%	2.7%	3.3%
	Exclusion words (>0 per Review) (Banerjee & Chua, 2017)	1	2	0	0	0	0	0	3	1

## Appendix D Review overview

**Table 8:** Review overview

Product	Review	Review Title	Review Text
Beer crate Attachment	Review 1	Great idea for any party.	The delivery even arrived two days earlier than predicted, high-quality wood, solid construction. I’ve use it as a side table apart from parties. The removable “tabletop” is unique and can be used as a tray.
	Review 2	Super cool piece!!!	Super cool item. Just as I had imagined it. Very good workmanship, fast shipping on the same day as the order. The beer crate attachment is very convenient to use. Even after half a year, there are no signs of wear. I am very satisfied. My guests were also thrilled with the tabletop attachment. Is a real eye-catcher. I can’t imagine living without one of those again.
	Review 3	Super Table!	It is easy to store and transport. The perfect solution for any party. Whether at home in front of the TV or in the garden. The idea with the insert in the middle is really great. This insert can be used as a tray. You get remove each bottle easily! A clear purchase recommendation.
Fan pack	Review 4	A definite purchase recommendation!!!	Very great products. Will order the set again as a gift. The cap fits perfectly. The cap fits perfectly. The Germany makeup holds up great. But it can also be washed off again easily. Shipping only took a day.
	Review 5	Fan Set Germany of the extra class!	Great fan pack. I am thrilled and recommend it without hesitation! We will have a lot of fun using it. And hopefully we’ll win:).
	Review 6	Let the World Cup begin.	Finally a fan pack with high quality contents. No cheap junk is offered here, but really high quality stuff. Both the cap and the lanyard, for example, can really be used not only during the World Cup. Let the World Cup begin because you are well equipped. I’m looking forward to a black-red-gold fan party!!!
Megaphone	Review 7	Simply brilliant and funny.	Perfectly suited for the World Cup. My grandchildren had a blast last week. And of course the adults, too. Would buy it again. The megaphone is really great for the price and is also a lot of fun.
	Review 8	Senseless but cool	I bought the megaphone for the Champions League final. I was the only one at the party who did not have a sore throat from screaming afterwards. A whole-hearted purchase recommendation without any regrets.
	Review 9	Really, what do you need such a megaphone for? Actually not at all! It is very easy to use	We drove Mallorca crazy with the megaphone during the World Cup. And it was really well suited for the evening. Great. Our neighbors also asked us to borrow it in the future.

The reviews in the vignette study were in German. The table provides the English translations. Appendix C was calculated based on the original German reviews.
